# Wood ducks and hooded mergansers as interspecific brood parasites: An evaluation of parasitic egg survival

**DOI:** 10.1002/ece3.11721

**Published:** 2024-07-11

**Authors:** Dylan L. Bakner, Kevin M. Ringelman, Larry A. Reynolds, Richard M. Kaminski, Scott E. Stephens, J. Brian Davis

**Affiliations:** ^1^ School of Renewable Natural Resources Louisiana State University Agricultural Center Baton Rouge Louisiana USA; ^2^ Louisiana Department of Wildlife and Fisheries Baton Rouge Louisiana USA; ^3^ Department of Wildlife Fisheries and Aquaculture Mississippi State University Mississippi State Mississippi USA; ^4^ Ducks Unlimited Inc. Memphis Tennessee USA

**Keywords:** *Aix sponsa*, dump nest, incubation, *Lophodytes cucullatus*, nesting, parasite, waterfowl

## Abstract

Conspecific and interspecific brood parasitism are alternate reproductive strategies more pervasive in waterfowl than in any other group of birds. While previous research has measured costs incurred by nest hosts incubating parasitized clutches, few studies have focused on the relative success of parasites. Here, we evaluated the success of wood duck (*Aix sponsa*) and hooded merganser (*Lophodytes cucullatus*) eggs laid parasitically in Louisiana and Mississippi. We monitored nest boxes, assigned eggs in each nest as host or parasitic, and determined the number of eggs that hatched and failed. Across all study areas (1994–1999 and 2020–2023), we monitored 1750 wood duck and 377 hooded merganser nests; ~13% of wood duck and ~24% of hooded merganser nests were interspecifically parasitized. We modeled egg survival of 2925 host and 691 parasitic eggs from 197 successful nests (≥1 hatched egg, regardless of species). Wood duck eggs laid in hooded merganser nests had lower survival [0.293, CI = 95% credible intervals (after, CI) = 0.176, 0.439] than hooded merganser eggs (0.762, CI = 0.704, 0.810) laid in wood duck nests. Clutch size negatively influenced parasitic wood duck egg survival (*β* = −.24, CI = −0.39, −0.10) but had a slight positive influence on parasitic hooded merganser eggs (*β* = .08, CI = 0.04, 0.12). Our results revealed that hooded merganser eggs experience higher success when laid parasitically in wood duck nests, whereas wood duck eggs experience lower success when laid parasitically in hooded merganser nests. Our results reveal new complexity in waterfowl interspecific brood parasitism, where the success of parasitic eggs is species‐, host‐, and context‐specific.

## Graphical abstract

We evaluated the relative success of wood ducks (*Aix sponsa*) and hooded mergansers (*Lophodytes cucullatus*) laying parasitically in Louisiana and Mississippi. Wood duck eggs laid in hooded merganser nests had lower survival than hooded merganser eggs laid in wood duck nests. Our results reveal new complexity in waterfowl nest parasitism, where nest parasites can incur costs that are species‐, host‐, and context‐specific.

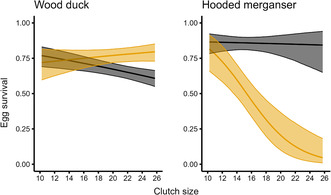

## 1 INTRODUCTION

Brood parasitism is an alternate reproductive strategy that is more pervasive in waterfowl than in any other group of birds (Lyon & Eadie, 2008; Yom‐Yov, 1980). Parasitic females lay their eggs in the nest of the same species, which is known as conspecific brood parasitism, or a different species, known as interspecific brood parasitism. This reproductive strategy used by many waterfowl species has resulted in some of the largest clutch sizes documented across all avian species (McCamant & Bolen, 1979; Semel et al., 1988). Researchers contend brood parasitism in waterfowl poses low costs to nest hosts as post‐hatch care is inexpensive as the young are nidifugous and precocial (Rohwer & Freeman, 1989).

While rearing parasitic young may be inexpensive to hosts, considerable costs may occur during the laying stage (Hepp et al., 1990), such as increased risk of nest abandonment when clutch size becomes too large (McCamant & Bolen, 1979; Semel et al., 1988; Sorenson, 1997). However, hosts will incubate supernormal clutches (McCamant & Bolen, 1979; Odell & Eadie, 2010; Rohwer, 1985), but they generally experience reduced hatchability (i.e., proportion of eggs hatching; McCamant & Bolen, 1979; Semel et al., 1988; Bakner et al., 2022) and prolonged incubation periods (Hope et al., 2021). While some studies have documented the negative consequences of brood parasitism to nest hosts (McCamant & Bolen, 1979; Péron & Koons, 2012), others found negligible effects (Hepp et al., 2020; Nielsen et al., 2006), and some suggest hosts may benefit from being parasitized because parasitic offspring dilute predation risk (Eadie & Lumsden, 1985; Robertson, 1998).

Understanding the costs of brood parasitism to hosts has received substantial scrutiny, but less attention has focused on the relative benefits accrued by females choosing to lay parasitically (Lyon & Eadie, 2008). In parasitized clutches, parasitic eggs encounter distinct challenges that could influence their overall success compared to host eggs (Eadie & Lyon, 2020; Sorenson, 1997). To ameliorate these costs, parasites have evolved to maximize the success of their parasitic eggs. For example, the eggshell strength of some species may enable stronger eggs to survive at higher rates in large clutches, where breakage is more common (Mallory & Weatherhead, 1990). This suggests that parasitic egg characteristics could influence egg success. However, a detailed examination of egg survival in mixed‐species clutches is lacking in waterfowl.

Here, we assess the relative success of wood ducks (*Aix sponsa*) and hooded mergansers (*Lophodytes cucullatus*) parasitizing the nests of each other in Louisiana and Mississippi. Although interspecific brood parasitism between these two species is common (Heusmann et al., 2000; Semel & Sherman, 1986; Zicus, 1990), the number of parasitic eggs each species lays in the nests of the other, as well as the success of these parasitic eggs, are poorly understood. Notably, hooded merganser eggshells are roughly three times stronger than wood duck eggs, which may influence egg survival rates (Gibson, 2022; Mallory & Weatherhead, 1990). In this study, we first described clutch composition (i.e., the number of host and parasitic eggs in the clutch) and then modeled the survival of eggs in mixed‐species clutches. We hypothesized that the survival of parasitic eggs would depend on clutch size, as hatchability decreases with increasing clutch size (McCamant & Bolen, 1979; Sorenson, 1997). We also hypothesized that parasitic wood duck and hooded merganser eggs would experience different survival rates, given the difference in eggshell strength (Gibson, 2022; Mallory & Weatherhead, 1990).

## 2 STUDY SPECIES

Wood ducks are widely distributed across North America and range from southern Canada to northern portions of Mexico (Baldassarre, 2014; Hepp & Bellrose, 2013). They are facultative conspecific and interspecific brood parasites that nest in tree cavities and artificial nest structures (Bellrose & Holm, 1994; Griscom, 1949; Heusmann et al., 2000). Wood ducks found at northern latitudes migrate south annually where females in the southeastern United States are year‐round residents. The difference in length of breeding season for these two regions is roughly 90 days, meaning the breeding season for southern areas can last up to 6 months. An individual wood duck lays approximately 10 eggs (Vrtiska, 1995) and incubation takes roughly 30 days to complete (Baldassarre, 2014).

Hooded mergansers are widely distributed across North America and range as far north as southern portions of Canada (Baldassarre, 2014; Dugger et al., 2009). Like wood ducks, hooded mergansers are facultative conspecific and interspecific brood parasites that nest in tree cavities and artificial nest structures (Bellrose & Holm, 1994; Griscom, 1949; Heusmann et al., 2000). Hooded mergansers found at northern latitudes make annual fall migrations while females in the southeastern United States are year‐round residents. Regardless of latitude, the hooded merganser breeding season lasts approximately 75 days (Baldassarre, 2014). An individual hooded merganser lays 10–13 eggs and incubation takes roughly 32 days to complete (Baldassarre, 2014; Morse & Wight, 1969).

## 3 STUDY AREA

### 3.1 Louisiana

We monitored existing nest boxes maintained by the Louisiana Department of Wildlife and Fisheries (after, LDWF) at Sherburne Wildlife Management Area (after, WMA), Thistlethwaite WMA, Indian Creek Reservoir, Lake Rodemacher, and Oden Lake in Louisiana 2020–2023; nest boxes were ~25 years old. Most nest boxes we monitored were duplex‐style, with two boxes mounted on either side of a pole (*N* = 190, 287, 245, and 245 boxes in 2020, 2021, 2022, and 2023, respectively) and some were single units (*N* = 76, 44, 31, and 31 boxes in 2020, 2021, 2022, and 2023, respectively). Sherburne WMA is in Iberville, Pointe Coupee, and St. Martin Parishes. The WMA is a bottomland hardwood forest with many backswamps and bayous and is located within the Atchafalaya floodplain. “North Farm” and “South Farm” are managed as moist soil impoundments on the eastern side of Sherburne WMA. Thistlethwaite is a bottomland hardwood forest in St. Landry parish that is privately owned but managed by LDWF. Indian Creek is located in Rapides Parish and surrounded by Alexander State Forest WMA. The WMA contains loblolly (*Pinus taeda*) and longleaf pine (*Pinus palustris*) stands and hardwoods that border creek drainages. Lake Rodemacher is in Rapides Parish and is used by the Brame Energy Center as a cooling resource when generating power. Oden Lake is in Rapides Parish, is privately owned, and is surrounded by a mixture of residential housing and cypress swamps.

### 3.2 Mississippi

In Mississippi, we collected data at the Sam D. Hamilton Noxubee and Yazoo National Wildlife Refuges (after, Noxubee and Yazoo) in the Interior Flatwoods and the Mississippi Alluvial Valley (i.e., Delta; Pettry, 1977). Overall, we monitored ~115 nest boxes annually at Noxubee from 1996 to 1999 and ~70 nest boxes at Yazoo from 1994 to 1997 (Davis et al., 1999). We erected nest boxes in Loakfoma Lake, Doyle Arm, and Bluff Lake at Noxubee during summer 1993 (Stephens et al., 1998). Dominant shoreline vegetation in Loakfoma Lake (243 ha) was willow (*Salix* spp.), scrub‐shrub (e.g., buttonbush; *Cephalanthus occidentalis*), and emergent vegetation (e.g., *Polygonum hydropiperoides*). Doyle Arm was a 16‐ha wetland with scattered smartweed (*Polygonum* spp.) and other herbaceous vegetation along its shoreline. Boxes in Bluff Lake (405 ha) were situated within a needle‐leaved, deciduous forest dominated by bald cypress (*Taxodium distichum*) and emergent vegetation as in Loakfoma Lake. At Yazoo NWR, boxes were erected in Deer Lake and Alligator Pond. Deer Lake (48 ha) was dominated by giant cut grass (*Zizaniopsis miliacea*) and giant water lily (*Nelumbo lutea*). Alligator Pond (28 ha) contained buttonbush, lotus (*Nelumbo nucifera*), and dead trees.

We monitored single‐unit nest boxes at Noxubee and Yazoo. All nest boxes were wooden and representative of dimensions of natural cavities used by these species (Davis et al., 1999; Denton et al., 2012; Stephens et al., 1998). The number of nest boxes monitored each year varied slightly with deterioration, windstorms, or flooding, and additional boxes were added in some years. Stephens et al. (1998) conducted an experiment at Noxubee and Yazoo NWRs to compare use, clutch sizes, and duckling production between small (internal dimensions: 25 × 13.75 × 42.5 cm) and large (internal dimensions: 25 × 25 × 60 cm) nest boxes, the former designed to reduce nesting space for parasitically laid eggs. Therefore, at each study site, half the nest boxes were small, and half were large.

## 4 METHODS

### 4.1 Field protocol

In Louisiana, we visited nest boxes at approximately 7‐day intervals to monitor egg and nest fates. We considered nests to be active if new eggs were added since the prior visit, egg incubation progressed (Weller, 1956), or by observing a bird incubating the clutch. We assigned all eggs from each nest a numeric ID by numbering the top of each egg (i.e., air‐cell‐end of egg) using a permanent marker and documented the species of each egg (Semel et al., 1988). We recorded eggs present or missing during weekly visits to nests. We concluded nests failed due to abandonment if we observed egg laying or incubation discontinue without sign of clutch loss between two consecutive visits. We determined nests failed due to a predation event when ≥1 egg went missing or was found depredated inside the nest box, causing egg laying or incubation to cease between two consecutive visits. We considered a clutch successful if it survived to hatch ≥1 egg, regardless of species. Following the termination of nests, we counted the number of eggs that failed or hatched. Eggs that were missing, unhatched, and non‐viable were declared failed; egg membranes were used to enumerate hatched eggs (Davis et al., 1998). Since the tops of the eggs typically remain intact after hatching, we utilized them to identify the species‐specific counts of hatched eggs. We determined the clutch size of each nest as the maximum number of eggs we observed in the clutch across all visits to the nest.

In Mississippi, boxes were checked monthly to meet specific research questions there (Davis et al., 1999, 2007, 2009). Eggs were not marked in Mississippi; thus, we did not have detailed information regarding egg failures. As a result, while we could distinguish between hatched and failed eggs, we could not diagnose specific causes of failure. We considered a clutch successful if it survived to hatch ≥1 egg; otherwise, the nest failed. Specific causes of nest failure were not documented in Mississippi.

We determined the host species (after, host) of each nest by observing the female incubating the clutch. We categorized clutches as being normal or mixed. Normal clutches were nests containing eggs belonging to a single species that corresponded to the nest host. Mixed clutches were nests that were interspecifically parasitized, and we categorized eggs as being host or parasitic. Host eggs were defined as those that matched the species incubating the clutch; others were classified as parasitic eggs. We determined eggs were laid by a wood duck if they were elliptically shaped and a creamy white color (Baldassarre, 2014); hooded merganser eggs were spherical and white (Mallory & Weatherhead, 1990). Given the prevalence of parasitism in both wood duck and hooded merganser populations, we recognize that conspecific brood parasitism was present in some normal clutches. However, limitations in our datasets prevented us from determining which nests were parasitized by conspecifics.

### 4.2 Analyses and model construction

We used data from both states to describe different characteristics of successful mixed clutches. We focused on analyses on nests that hatched >1 egg because diagnosing differential causes of species‐specific egg survival is not possible in clutches where all eggs failed to survive. We predicted clutch size, the number of parasitic eggs, and the number of hatched parasitic eggs using multilevel Poisson models:

$$c_{i}\sim \text{Poisson}\left(\lambda_{i}\right),$$

$$\begin{array}{cc} \log\left(\lambda_{i}\right)=\; & \alpha +\beta_{1}\times {\text{nest host}}_{i}+\beta_{2}\times \mathrm{box}\;{\text{size}}_{i}+\beta_{3}\\ & \times {\left(\text{nest}\;{\text{host}}_{i}\times \mathrm{box}\;{\text{size}}_{i}\right)}_{+}\;\varepsilon_{i}, \end{array}$$

where (*c*_*i*_) is the observed count of each response variable for nest *i*. We used the log link function to evaluate the relationship between our fixed effects and the expected count (*λ*_*i*_). We considered an interaction between nest host and box size (small vs. large) as our fixed effect and included nest ID as the random effect (*ε*_*i*_) in each model. We included box size in our model because Stephens et al. (1998) found this variable influenced the total clutch size of wood duck nests and the number of hatched eggs. All nest boxes in Louisiana were assigned to the large box category. We estimated egg survival (i.e., the probability an egg hatches) for host and parasitic eggs as a binomial outcome:

$$y_{i,j}\sim \text{Binomial}\left(n_{i,j},\Phi_{i,j}\right),$$

$$\begin{array}{c} \text{logit}\left(\Phi_{i,j}\right)\sim \beta_{0}+\beta_{1}\times {\text{host}}_{i,j}+\beta_{2}\times {\text{clutch size}}_{i,j}+\beta_{3}\\ \times \left({\text{host}}_{i,j}\times {\text{clutch size}}_{i,j}\right)+\beta_{4}\times \mathrm{box}\;{\text{size}}_{i,j}+\varepsilon_{i}, \end{array}$$

where *y* is egg survival for nest *i* and egg type *j*. For each nest *i* and egg type *j*, we considered the total number of eggs as the number of trials (*n*), with the success probability (*Φ*) as the number of eggs that hatched. We considered nest host, clutch size, and box size as fixed effects and nest ID as a random effect in our models. We also considered the interaction between nest host and clutch size.

To further evaluate the success of parasitic eggs laid in nests with varying numbers of host eggs, we predicted the number of host and parasitic eggs hatching from different clutches, using the model coefficients from our top egg survival model. In the first illustrative scenario, we held the number of host eggs constant at 10 while varying the number of parasitic eggs (after, constant mixed clutch scenario). In the second scenario, the number of host and parasitic eggs was proportional, while the overall clutch size varied (after, proportional mixed clutch scenario).

We fit our Bayesian multilevel models in Program R version 4.2.2 (R Core Team, 2023) using package brms (Bürkner, 2017). We ran 4 Markov Chain Monte Carlo (after, MCMC) chains of 20,000 iterations, discarding 500 samples during the warm‐up period, saving every tenth iteration, and used uninformative prior distributions for fixed and random effects. We ensured MCMC chains converged by examining trace plots and by using the Gelman‐Rubin statistic ($\hat{R}$ value <1.05; Gelman & Rubin, 1992). Our egg survival model was compared to a null model by calculating the percentage of variation explained by fixed effects using Grosbois et al. (2008) seventh equation:

$$\begin{array}{c} \frac{{\hat{\sigma }}^{2}\left(\text{random}-\text{effects}-\text{only model}\right)-{\hat{\sigma }}^{2}\left(\text{fixed effects model}\right)}{\;{\hat{\sigma }}^{2}\left(\text{random}-\text{effects}-\text{only model}\right)} \end{array}$$

For all models, 95% credible intervals (after, CI) that did not overlap zero indicated significant effects (Data S1).

Research at the Louisiana study sites was conducted under U.S. Fish and Wildlife Service banding permit #06669 and Special Use Permit 43614‐20‐04; Louisiana Department of Wildlife and Fisheries state collecting permits WDP‐20‐037 and WDP‐21‐060, and Wildlife Management Area Permit WL‐Research‐2020‐03; Louisiana State University Institutional Animal Care and Use Protocol (after, IACUC) A2019‐27. Research activities in Mississippi were approved by Mississippi State University IACUC Protocol No. 96‐018.

## 5 RESULTS

We studied 2127 nests that were hosted by 1750 wood ducks and 377 hooded mergansers (Table 1). A total of 228 (13.0%) wood ducks and 90 (23.9%) hooded merganser nests contained mixed clutches. We monitored 25,860 wood ducks and 5707 hooded merganser eggs. A total of 11,948 (46.2%) wood ducks and 2944 (51.6%) merganser eggs hatched. For wood ducks, 10,330 (86.5%) eggs hatched from 936 normal clutches, 1532 (12.8%) wood ducks, and 388(13.2%) merganser eggs hatched from 147 mixed wood duck clutches. For mergansers, 2143 (72.8%) eggs hatched from 180 normal clutches, and 463 (15.7%) merganser and 86 (0.7%) wood duck eggs hatched from 50 mixed merganser clutches.

**TABLE 1:** Number of wood duck (*Aix sponsa*) and hooded merganser (*Lophodytes cucullatus*) nests monitored and successful (%) in Louisiana (2020–2023) and Mississippi (1996–1999) by clutch type.

	Louisiana				Mississippi			
	Wood duck		Hooded merganser		Wood duck		Hooded merganser	
Nests	Normal	Mixed	Normal	Mixed	Normal	Mixed	Normal	Mixed
Total	1103	119	168	68	419	109	119	22
Successful	543 (49%)	52 (44%)	74 (44%)	33 (49%)	393 (94%)	95 (87%)	106 (89%)	17 (77%)

*Note*: Normal clutches were nests containing eggs belonging to a single species that corresponded to the nest host. Mixed clutches were nests that were interspecifically parasitized.

We considered 2925 host and 691 parasitic eggs from 197 successful mixed clutches in our analyses. We found mixed wood duck clutches were larger (20.3 eggs, CI = 19.3, 21.3) than mixed merganser clutches (14.7 eggs, CI = 13.6, 16.0; *β* = −.32, CI = −0.41, −0.23). Mixed clutches in small boxes contained fewer eggs for each nest host (wood duck 15.0, CI = 13.6, 16.7; hooded merganser 9.2, CI = 6.7, 12.6; *β* = −.30, CI = −0.41, −0.19) compared to large boxes (wood duck 20.3, CI = 19.3, 21.3; hooded merganser 14.7, CI = 13.6, 16.0). The mean number of parasitic eggs in mixed clutches was similar for nest hosts (wood duck 3.5, CI = 3.0, 4.0; hooded merganser 3.0, CI = 2.4, 3.7; *β* = −.14, CI = −0.40, 0.11). Fewer parasitic eggs were in smaller boxes (wood duck 2.1, CI = 1.5, 2.8; hooded merganser 2.1, CI = 0.9, 4.6; *β* = −.51, CI = −0.83, −0.19) compared to larger boxes (wood duck 3.5, CI = 3.0, 4.0; hooded merganser 3.0, CI = 2.4, 3.7). The mean number of parasitic ducklings hatched from mixed clutches was greater for nests hosted by wood ducks (2.3, CI = 1.9, 2.7) compared to those hosted by hooded mergansers (1.3, CI = 0.9, 1.8; *β* = −.57, CI = −0.94, −0.22).

**FIGURE 1:**
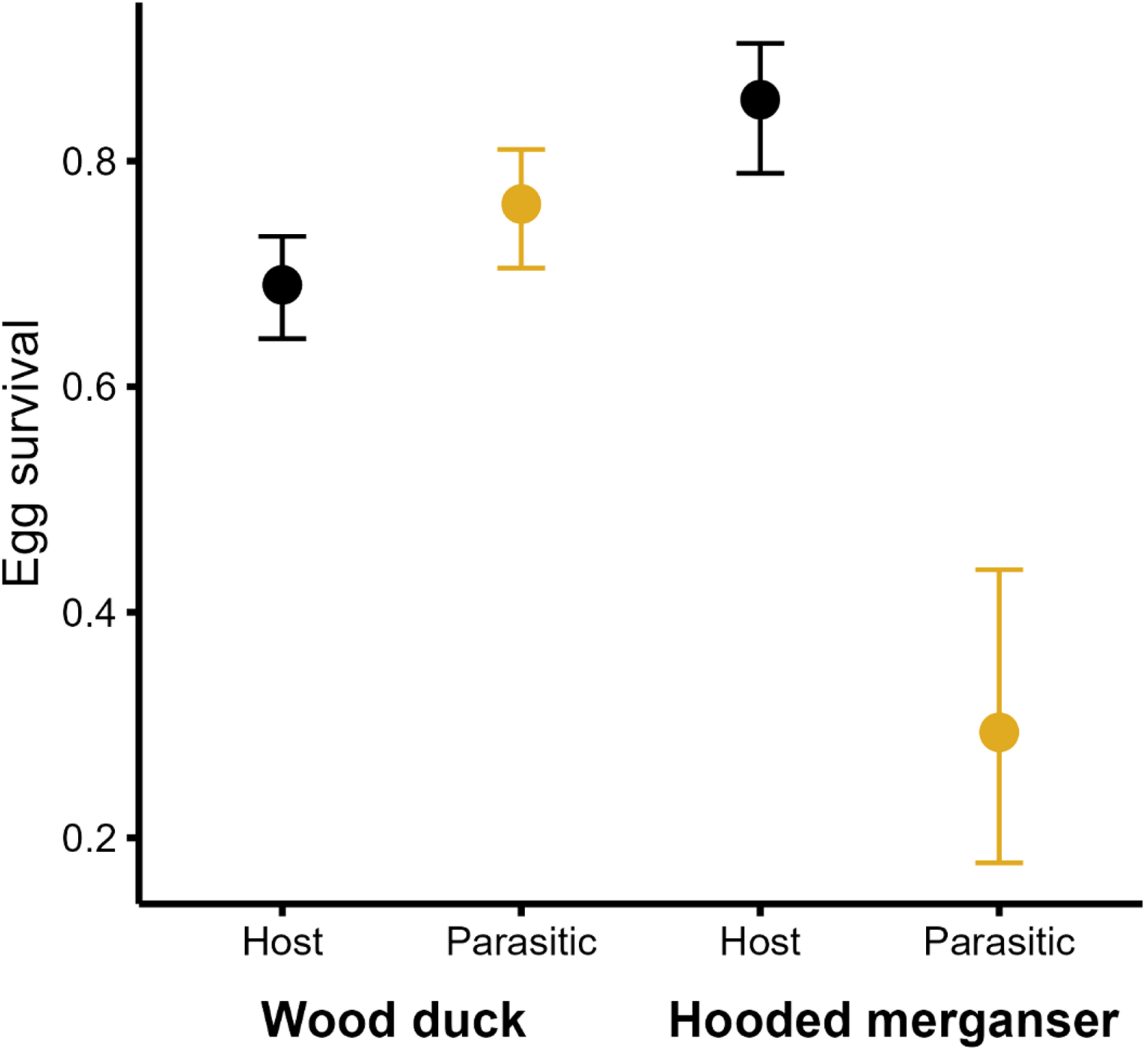
Model predictions of egg survival for wood ducks (*Aix sponsa*) and hooded mergansers (*Lophodytes cucullatus*). The bolded species name indicates the nest host (i.e., species incubating). Host eggs were those that matched the species incubating the clutch (black), including eggs that were laid parasitically by conspecifics; all others were parasitic eggs (gold).

**FIGURE 2:**
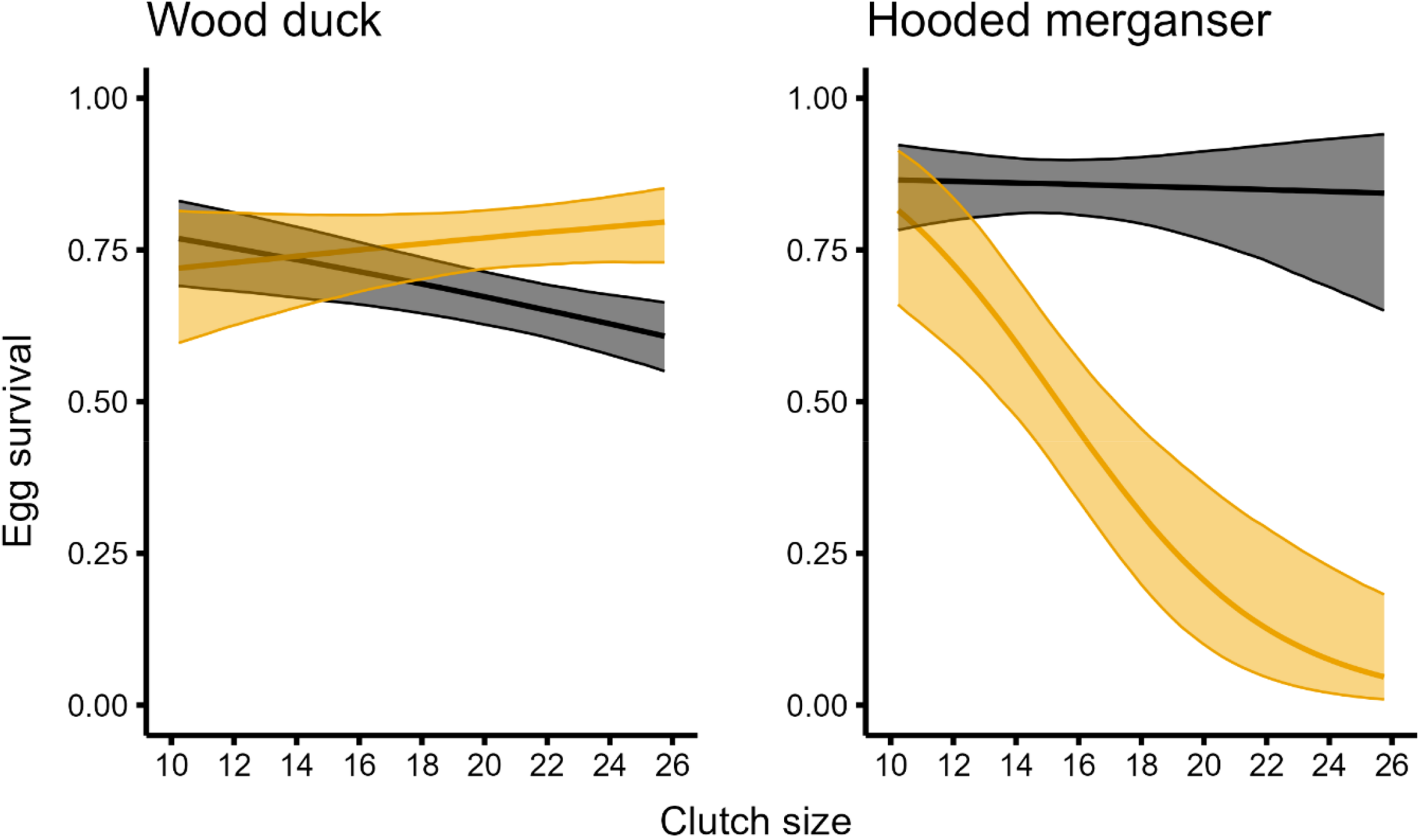
Model predictions (with 95% credible intervals; after, CI) of egg survival across different clutch sizes for wood ducks (*Aix sponsa*) and hooded mergansers (*Lophodytes cucullatus*). Host eggs (gray CIs) were those that matched the species incubating the clutch, including eggs that were laid parasitically by conspecifics; all others were parasitic eggs belonging to the other species (gold CIs).

Egg survival was the same for most egg types (host wood duck eggs: 0.690, CI = 0.644, 0.735; host hooded merganser eggs: 0.854, CI = 0.789, 0.904; parasitic hooded merganser eggs: 0.762, CI = 0.704, 0.810), except for parasitic wood duck eggs laid in hooded merganser nests, which had substantially lower survival (0.293, CI = 0.176, 0.439; *β* = −1.05, CI = −1.95, −0.16; Figure 1). Clutch size had a negative effect on egg survival (*β* = −0.05, CI = −0.08, −0.02). The interaction term had a negative effect on the survival of parasitic wood duck eggs (*β* = −.24, CI = −0.39, −0.10) and a slightly positive influence on parasitic hooded merganser eggs (*β* = .08, CI = 0.04, 0.12; Figure 2). Our fixed effects explained only 11.5% of the variation in egg survival observed between nests.

**FIGURE 3:**
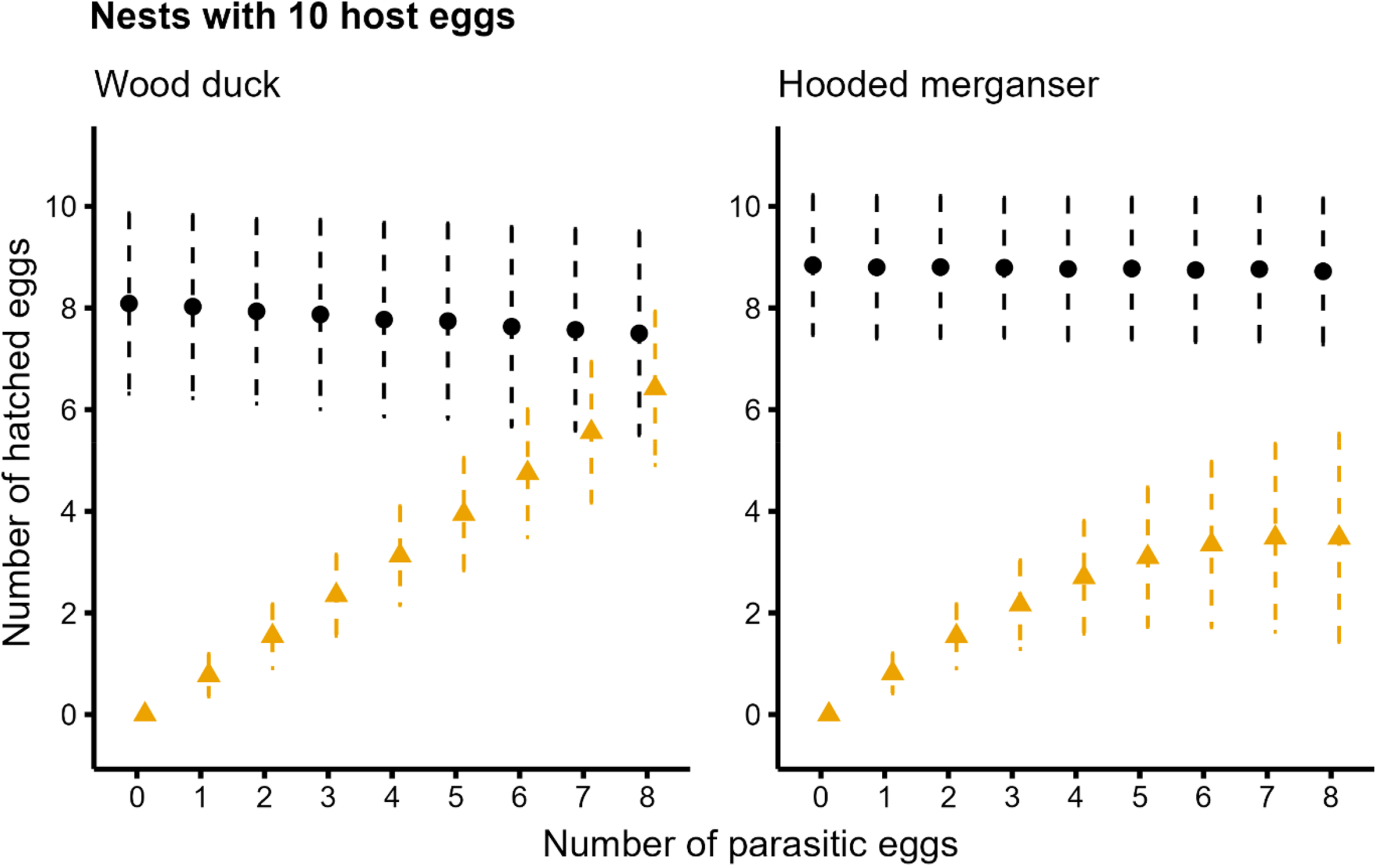
Model predictions (with 95% credible intervals; after, CI) showing the number of wood duck (*Aix sponsa*) and hooded merganser (*Lophodytes cucullatus*) eggs expected to hatch from mixed clutches (i.e., interspecifically parasitized). The number of host eggs is held constant at 10. Host eggs (black circles and CIs) were those that matched the species incubating the clutch, including eggs that were laid parasitically by conspecifics; all others were parasitic eggs belonging to the other species (gold triangles and CIs).

**FIGURE 4:**
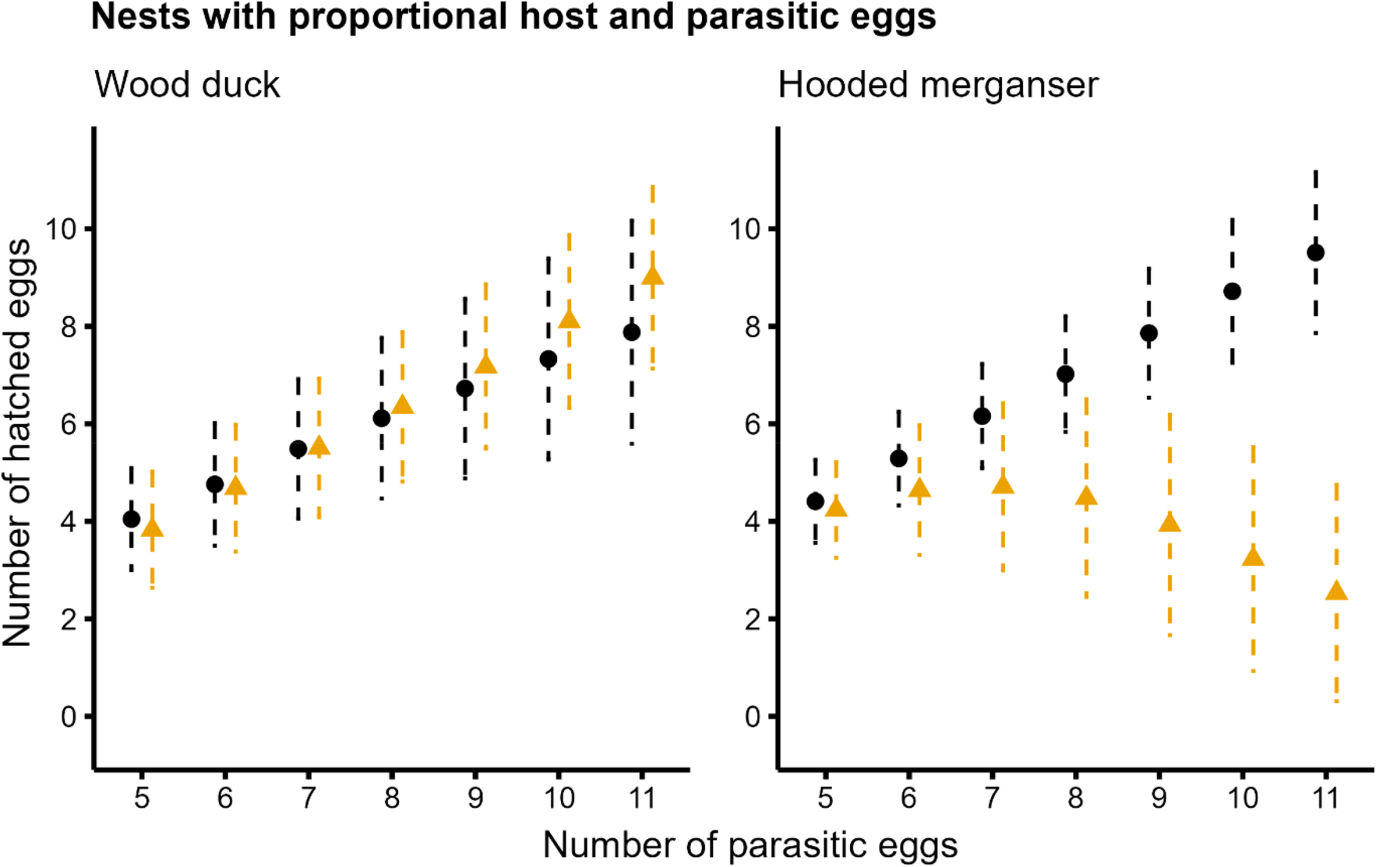
Model predictions (with 95% credible intervals; after, CI) showing the number of wood duck (*Aix sponsa*) and hooded merganser (*Lophodytes cucullatus*) eggs expected to hatch from mixed clutches (i.e., interspecifically parasitized). The number of host eggs is proportional to the number of parasitic eggs. Host eggs (black circles and CIs) were those that matched the species incubating the clutch, including eggs that were laid parasitically by conspecifics; all others were parasitic eggs belonging to the other species (gold triangles and CIs).

In both the constant mixed clutch and proportional mixed clutch scenarios, parasitic hooded merganser eggs were more successful than wood duck eggs. The number of hatched parasitic hooded merganser eggs continued to increase as more parasitic eggs were laid, even at large clutch sizes (Figure 3). In contrast, parasitic wood duck eggs were less successful than host hooded merganser eggs, where hatching more than three parasitic eggs was unlikely under the constant mixed clutch (Figure 3). Parasitic wood duck eggs were most successful when laid in smaller hooded merganser clutches under the proportionally mixed clutch scenario (Figure 4); however, when the clutch size exceeded 12 eggs (six hosts and six parasitics), the proportion of parasitic wood duck eggs hatching decreased (Figure 4).

## 6 DISCUSSION

Mixed clutches of wood duck and hooded merganser eggs occur in several regions of North America (Doty et al., 1984; Kennamer et al., 1988; Mallory, 2003), and the frequency and success of mixed clutches reported in previous studies are comparable to our findings (Doty et al., 1984; Mallory et al., 2002). In our study, 13.0% of wood duck nests and 23.9% of hooded merganser nests contained mixed clutches. For these clutches, 64.5% hosted by wood ducks and 55.6% hosted by hooded mergansers were successful. Overall, we observed higher success for hooded mergansers parasitizing wood duck nests as the survival of their parasitic eggs remained high across all clutch sizes. Furthermore, clutch composition had little influence on the fate of parasitic hooded merganser eggs. Conversely, we observed lower success for parasitic wood duck eggs laid in hooded merganser nests, where egg survival declined precipitously with greater clutch size and was also influenced by clutch composition.

Wood duck eggs survived at lower rates when laid parasitically in hooded merganser nests, which may have several explanations. A plausible explanation is that the shells of wood duck eggs are thinner and structurally weaker than those of hooded mergansers (Gibson, 2022). Accordingly, wood duck eggs may be more likely to crack in hooded merganser nests, and the damaged eggs are subsequently removed by the host (Bakner & Ringelman, 2023; Dugger et al., 1999). Dugger et al. (1999) conducted experiments using urethane to coat a subset of wood duck eggs to enhance durability. Urethane‐treated wood duck eggs were less likely to be removed from the nest box than untreated eggs. These results were consistent with Eadie's (1989) hypothesis that egg removal is a response to broken eggs in a nest and not an antiparasitic behavior exhibited by a host. Eggs may crack in the nest for various reasons, and at our Louisiana study sites the frequency of cracked eggs was unusually high because of frequent partial clutch depredation by red‐bellied woodpeckers (*Melanerpes carolinus*; Bakner & Ringelman, 2023). Our observations of trail camera videos recorded inside nest boxes show that woodpeckers often fail to peck and fracture black‐bellied whistling‐duck (*Dendrocygna atumnailis*) eggs (Bakner et al. personal observations), whose eggshell strength is intermediate to wood ducks and mergansers (Gibson, 2022). Thus, we speculate that wood duck eggs, with their thinner and weaker eggshells, may be depredated at greater rates compared to hooded merganser eggs. Moreover, clutch size alone may influence the rates at which wood duck eggs are broken and removed from the nest, as wood duck egg survival decreased with clutch size, both in normal nests and parasitic nests (Figure 2).

Stronger eggshells likely benefit hooded merganser eggs but this may not be the sole factor explaining their high survival when laid parasitically in wood duck nests. We advocate that the timing of egg‐laying is also important. Our field observations from Louisiana and Mississippi study sites indicate that most mixed wood duck clutches receive parasitic hooded merganser eggs prior to incubation. Odell and Eadie (2010) found that wood ducks selected smaller clutches to host their parasitic eggs, potentially perceiving them as in the laying stage. Likewise, hooded mergansers could potentially use host nest clutch sizes as a cue for gauging the nesting stage before laying parasitically. This behavior could boost their egg survival by avoiding nests already being incubated, which would reduce the probability of their eggs hatching incubatory asynchrony (Morse & Wight, 1969).

Our results and observations herein are supported by a previous study in Missouri, where nesting efficiency (eggs laid per ducklings hatched) for merganser eggs incubated by wood ducks was 0.56 and 0.46 for wood duck eggs incubated by hooded mergansers (Lemons, 2004). Hooded mergansers laid 444 eggs in wood duck nests and 251 (57%) parasitic ducklings exited nest boxes which accounted for ~14% of all hooded merganser production observed during the three‐year study. During the same study, wood ducks laid 91 eggs in hooded merganser nests with only 15 (16%) parasitic ducklings exiting nest boxes. Hooded mergansers may have constraints in effectively incubating large clutches, or actively rearranging nest contents to prioritize effective incubation of their own eggs. Future experiments may be able to better diagnose behavioral responses to parasitic cues: for example, manipulation of egg numbers might offer valuable insight into the preferred wood duck clutch sizes targeted by parasitic hooded mergansers (Odell & Eadie, 2010). Furthermore, laboratory‐ or aviary‐based studies could explore the relative tolerance of wood duck and merganser eggs to imperfect incubation conditions (DuRant et al., 2010). It should also be noted that physical characteristics of the box can affect rates of parasitism: Stephens et al. (1998) found lower rates of parasitism in half‐volume small boxes, but more ducklings ultimately left standard boxes which accommodated larger clutches.

Our findings provide baseline information on the success of laying parasitically, which may have important ramifications for nest box programs. We also recommend future consideration of the effects of conspecific parasitism, which would provide a more comprehensive view of the costs of parasitism. Achieving this would require using genetic techniques to better assign eggs to nest hosts and parasites (Eadie et al., 2010; Lemons et al., 2011; Pöysä et al., 2009). To understand the lower survival of parasitic wood duck eggs in hooded merganser nests, it will be important for future studies to conduct weekly nest box visits and keep detailed records of egg losses, such as through the use of trail cameras, to confirm some of our speculations. One important conclusion from our study is that hooded merganser eggs are more successful when laid parasitically in wood duck nests, while wood duck eggs are less successful when laid parasitically in hooded merganser nests. Future research should assess the combined effects of conspecific and interspecific brood parasitism to achieve a full understanding of the success of host and parasitic eggs.

## AUTHOR CONTRIBUTIONS

**Dylan L. Bakner:** Conceptualization (equal); data curation (equal); formal analysis (equal); investigation (equal); visualization (equal); writing – original draft (equal); writing – review and editing (equal). **Kevin M. Ringelman:** Conceptualization (equal); data curation (equal); formal analysis (equal); project administration (equal); resources (equal); supervision (equal); writing – original draft (equal); writing – review and editing (equal). **Larry A. Reynolds:** Funding acquisition (equal); methodology (equal); project administration (equal); resources (equal); supervision (equal). **Richard M. Kaminski:** Conceptualization (equal); data curation (equal); funding acquisition (equal); project administration (equal); resources (equal); writing – review and editing (equal). **Scott E. Stephens:** Data curation (equal); funding acquisition (equal); investigation (equal); project administration (equal); resources (equal); writing – review and editing (equal). **J. Brian Davis:** Conceptualization (equal); funding acquisition (equal); investigation (equal); methodology (equal); project administration (equal); resources (equal); supervision (equal); writing – review and editing (equal).

## CONFLICT OF INTEREST STATEMENT

None.

## Supporting information


Data S1:

## Data Availability

Data and code to reproduce the analysis are available to reviewers and will remail available to viewers following the acceptance of this article.

## References

[ece311721-bib-0001] Bakner, D. L. , Miranda, K. E. , & Ringelman, K. M. (2022). Louisiana Black‐bellied Whistling‐Duck clutch characteristics in the presence of conspecific and interspecific brood parasitism. Journal of Field Ornithology, 93. 10.5751/JFO-00184-930408

[ece311721-bib-0002] Bakner, D. L. , & Ringelman, K. M. (2023). A simple trail camera modification reveals red‐bellied woodpeckers as important egg predators of box‐nesting wood ducks. Food Webs, 35, e00283. 10.1016/j.fooweb.2023.e00283

[ece311721-bib-0003] Baldassarre, G. (2014). Ducks, geese, and swans of North America. John Hopkins University Press.

[ece311721-bib-0004] Bellrose, F. C. , & Holm, D. J. (1994). Ecology and management of the wood duck. Stackpole Books.

[ece311721-bib-0005] Bürkner, P.‐C. (2017). brms: An R package for Bayesian multilevel models using Stan. Journal of Statistical Software, 80, 1–28. 10.18637/jss.v080.i01

[ece311721-bib-0006] Davis, J. B. , Cox, R. R., Jr. , Kaminski, R. M. , & Leopold, B. D. (2007). Survival of wood duck ducklings and broods in Mississippi and Alabama. Journal of Wildlife Management, 71, 507–517.

[ece311721-bib-0007] Davis, J. B. , Kaminski, R. M. , & Stephens, S. E. (1998). Wood duck egg‐shell membranes predict duckling numbers. Wildlife Society Bulletin, 26, 299–301.

[ece311721-bib-0008] Davis, J. B. , Leopold, B. D. , Kaminski, R. M. , & Cox, R. R., Jr. (2009). Wood Duck duckling mortality and habitat implications in floodplain systems. Wetlands, 29, 607–614.

[ece311721-bib-0009] Davis, J. B. , Stephens, S. E. , Leopold, B. D. , Kaminski, R. M. , & Gerard, P. D. (1999). Wood duck reproduction in small and large nest boxes in Mississippi: A continued experiment. Proceedings of the Annual Conference of the Southeastern Association of Fish and Wildlife Agencies, 53, 257–269.

[ece311721-bib-0010] Denton, J. C. , Roy, C. L. , Soulliere, G. J. , & Potter, B. A. (2012). Change in density of duck nest cavities at forests in the north central United States. Journal of Fish and Wildlife Management, 3, 76–88. 10.3996/112011-JFWM-067

[ece311721-bib-0011] Doty, H. A. , Lee, F. B. , Kruse, A. D. , Matthews, J. W. , Foster, J. R. , & Arnold, P. M. (1984). Wood duck and hooded merganser nesting on Arrowwood NWR, North Dakota. The Journal of Wildlife Management, 48, 577–580. 10.2307/3801193

[ece311721-bib-0012] Dugger, D. B. , Bollmann, L. C. , & Fredrickson, L. H. (1999). Response of female hooded mergansers to eggs of an interspecific brood parasite. The Auk, 116, 269–273.

[ece311721-bib-0013] Dugger, D. B. , Dugger, M. K. , & Fredrickson, L. H. (2009). Birds of the world. Cornell Laboratory of Ornithology.

[ece311721-bib-0014] DuRant, S. , Hepp, G. , Moore, I. , Hopkins, B. , & Hopkins, W. (2010). Slight differences in incubation temperature affect early growth and stress endocrinology of wood duck (*Aix sponsa*) ducklings. Journal of Experimental Biology, 213, 45–51. 10.1242/jeb.034488 20008361

[ece311721-bib-0015] Eadie, J. M. (1989). Alternative reproductive tactics in a precocial bird: The ecology and evolution of brood parasitism in goldeneyes. (Ph.D. Dissertation). University of British Columbia.

[ece311721-bib-0016] Eadie, J. M. , & Lumsden, H. G. (1985). Is nest parasitism always deleterious to goldeneyes? The American Naturalist, 126, 859–866. 10.1086/284458

[ece311721-bib-0017] Eadie, J. M. , & Lyon, B. E. (2020). Environmentally driven escalation of host egg rejection decimates success of an avian brood parasite. Behavioral Ecology, 31, 1316–1325. 10.1093/beheco/araa084

[ece311721-bib-0018] Eadie, J. M. , Smith, J. N. , Zadworny, D. , Kühnlein, U. , & Cheng, K. (2010). Probing parentage in parasitic birds: An evaluation of methods to detect conspecific brood parasitism using goldeneyes *Bucephala islandica* and *Bl. clangula* as a test case. Journal of Avian Biology, 41, 163–176. 10.1111/j.1600-048X.2009.04735.x

[ece311721-bib-0019] Gelman, A. , & Rubin, D. B. (1992). Inference from iterative simulation using multiple sequences. Journal of Statistical Science, 7, 457–472. 10.1214/ss/1177011136

[ece311721-bib-0020] Gibson, J. T. (2022). Nesting ecology of wood ducks and other cavity‐nesting ducks in Mississippi. (M.S. Thesis). Mississippi State University.

[ece311721-bib-0021] Griscom, L. (1949). The birds of Concord. Harvard University Press.

[ece311721-bib-0022] Grosbois, V. , Gimenez, O. , Gaillard, J.‐M. , Pradel, R. , Barbraud, C. , Clobert, J. , Møller, A. P. , & Weimerskirch, H. (2008). Assessing the impact of climate variation on survival in vertebrate populations. Biological Reviews, 83, 357–399. 18715402 10.1111/j.1469-185X.2008.00047.x

[ece311721-bib-0023] Hepp, G. R. , & Bellrose, F. C. (2013). Birds of the world. Cornell Laboratory of Ornithology.

[ece311721-bib-0024] Hepp, G. R. , Gitzen, R. A. , & Kennamer, R. A. (2020). Relative importance of vital rates to population dynamics of wood ducks. The Journal of Wildlife Management, 84, 320–330. 10.1002/jwmg.21792

[ece311721-bib-0025] Hepp, G. R. , Kennamer, R. A. , & Harvey, W. F. (1990). Incubation as a reproductive cost in female wood ducks. Auk, 107, 756–764.

[ece311721-bib-0026] Heusmann, H. W. , Early, T. , & Nikula, B. (2000). Evidence of an increasing hooded merganser population in Massachusetts. The Wilson Bulletin, 112, 413–415. 10.1676/0043-5643

[ece311721-bib-0027] Hope, S. F. , DuRant, S. E. , Hallagan, J. J. , Beck, M. L. , Kennamer, R. A. , & Hopkins, W. A. (2021). Incubation temperature as a constraint on clutch size evolution. Functional Ecology, 35, 909–919. 10.1111/1365-2435.13764

[ece311721-bib-0028] Kennamer, R. A. , Harvey, W. F. , & Hepp, G. R. (1988). Notes on hooded merganser nests in the coastal plain of South Carolina. The Wilson Bulletin, 100, 686–688.

[ece311721-bib-0029] Lemons, P. R. (2004). Factors affecting wood duck and hooded merganser brood survival. University of Missouri.

[ece311721-bib-0030] Lemons, P. R. , Sedinger, J. S. , & Svete Randle, P. (2011). Detecting conspecific brood parasitism using egg morphology in black brant *Branta bernicla nigricans* . Journal of Avian Biology, 42, 282–288. 10.1111/j.1600-048X.2011.05217.x

[ece311721-bib-0031] Lyon, B. E. , & Eadie, J. M. (2008a). Conspecific brood parasitism in birds: A life‐history perspective. Annual Review of Ecology, Evolution, and Systematics, 39, 343–363. 10.1146/annurev.ecolsys.39.110707.173354

[ece311721-bib-0032] Mallory, M. (2003). Partial clutch loss in wood ducks *Aix sponsa* nesting near Ottawa, Canada. Wild, 54, 63–70.

[ece311721-bib-0033] Mallory, M. L. , Taverner, A. , Bower, B. , & Crook, D. (2002). Wood duck and hooded merganser breeding success in nest boxes in Ontario. Wildlife Society Bulletin, 30, 310–316.

[ece311721-bib-0034] Mallory, M. L. , & Weatherhead, P. J. (1990). Effects of nest parasitism and nest location on eggshell strength in waterfowl. The Condor, 92, 1031–1039.

[ece311721-bib-0035] McCamant, R. E. , & Bolen, E. G. (1979). A 12‐year study of nest box utilization by black‐bellied whistling ducks. The Journal of Wildlife Management, 43, 936–943. 10.2307/3808277

[ece311721-bib-0036] Morse, T. E. , & Wight, H. M. (1969). Dump nesting and its effect on production in wood ducks. The Journal of Wildlife Management, 33, 284–293. 10.2307/3799828

[ece311721-bib-0037] Nielsen, C. L. R. , Gates, R. J. , & Parker, P. G. (2006). Intraspecific nest parasitism of wood ducks in natural cavities: Comparisons with nest boxes. The Journal of Wildlife Management, 70, 835–843.

[ece311721-bib-0038] Odell, N. S. , & Eadie, J. M. (2010). Do wood ducks use the quantity of eggs in a nest as a cue to the nest's value? Behavioral Ecology, 21, 794–801. 10.1093/beheco/arq055

[ece311721-bib-0039] Péron, G. , & Koons, D. N. (2012). Integrated modeling of communities: Parasitism, competition, and demographic synchrony in sympatric ducks. Ecology, 93, 2456–2464. 10.1890/11-1881.1 23236916

[ece311721-bib-0040] Pettry, D. E. (1977). Soil resource areas of Mississippi. Department of Agronomy, Mississippi Agricultural Forestry Experiment Station, information sheet number 1278, Mississippi State University.

[ece311721-bib-0041] Pöysä, H. , Lindblom, K. , Rutila, J. , & Sorjonen, J. (2009). Reliability of egg morphology to detect conspecific brood parasitism in goldeneyes *Bucephala clangula* examined using protein fingerprinting. Journal of Avian Biology, 40, 453–456. 10.1111/j.1600-048X.2008.04528.x

[ece311721-bib-0042] R Core Team . (2023). R: A language and environment for statistical computing. R Foundation for Statistical Computing.

[ece311721-bib-0043] Robertson, G. J. (1998). Egg adoption can explain joint egg‐laying in common eiders. Behavioral Ecology and Sociobiology, 43, 289–296. 10.1007/s002650050493

[ece311721-bib-0044] Rohwer, F. C. (1985). The adaptive significance of clutch size in prairie ducks. The Auk, 102, 354–361.

[ece311721-bib-0045] Rohwer, F. C. , & Freeman, S. (1989). The distribution of conspecific nest parasitism in birds. Canadian Journal of Zoology, 67, 239–253. 10.1139/z89-035

[ece311721-bib-0047] Semel, B. , & Sherman, P. W. (1986). Dynamics of nest parasitism in wood ducks. The Auk, 103, 813–816.

[ece311721-bib-0048] Semel, B. , Sherman, P. W. , & Byers, S. M. (1988). Effects of brood parasitism and nest‐box placement on wood duck breeding ecology. The Condor, 90, 920–930. 10.2307/1368849

[ece311721-bib-0049] Sorenson, M. D. (1997). Effects of intra‐and interspecific brood parasitism on a precocial host, the canvasback, *Aythya valisineria* . Behavioral Ecology, 8, 153–161. 10.1093/beheco/8.2.153

[ece311721-bib-0050] Stephens, S. E. , Kaminski, R. M. , Leopold, B. D. , & Gerard, P. D. (1998). Reproduction of wood ducks in small and large nest boxes. Wildlife Society Bulletin, 26, 159–167.

[ece311721-bib-0051] Vrtiska, M. P. (1995). Aspects of reproductive and remige molt biology in wood ducks. (Ph.D. Dissertation). Mississippi State University.

[ece311721-bib-0052] Weller, M. W. (1956). A simple field candler for waterfowl eggs. The Journal of Wildlife Management, 20, 111–113. 10.2307/3797414

[ece311721-bib-0053] Yom‐Yov, Y. (1980). Intraspecific nest parasitism in birds. Biological Reviews, 55, 93–108. 10.1111/j.1469-185X.1980.tb00689.x

[ece311721-bib-0054] Zicus, M. C. (1990). Nesting biology of hooded mergansers using nest boxes. The Journal of Wildlife Management, 54, 637–643. 10.2307/3809362

